# Supplementary material for: Pyruvate Dehydrogenase Kinase 1 inhibition mediated oxidative phosphorylation enhancement in cartilage promotes osteoarthritis progression
Source: BMC Musculoskelet Disord. 2023 Jul 20;24:597. doi: 10.1186/s12891-023-06585-6 (PMC10357736; doi:10.1186/s12891-023-06585-6)
Supplement: Supplementary file 1 — Additional file 1. [file 12891_2023_6585_MOESM1_ESM.zip › BP.Gsea.1653623667859/GOBP_MEMBRANE_LIPID_CATABOLIC_PROCESS.html]

Details for gene set GOBP\_MEMBRANE\_LIPID\_CATABOLIC\_PROCESS[GSEA]

|  || Dataset | 55457\_collapsed\_to\_symbols.cls.cls #OA\_versus\_normal.cls.cls #OA\_versus\_normal\_repos |
| Phenotype | cls.cls#OA\_versus\_normal\_repos |
| Upregulated in class | OA |
| GeneSet | GOBP\_MEMBRANE\_LIPID\_CATABOLIC\_PROCESS |
| Enrichment Score (ES) | 0.45792273 |
| Normalized Enrichment Score (NES) | 1.623758 |
| Nominal p-value | 0.004705882 |
| FDR q-value | 0.22967611 |
| FWER p-Value | 1.0 |
Table: GSEA Results Summary

  

Fig 1: Enrichment plot: GOBP\_MEMBRANE\_LIPID\_CATABOLIC\_PROCESS      
 Profile of the Running ES Score & Positions of GeneSet Members on the Rank Ordered List

  

| SYMBOL | TITLE | RANK IN GENE LIST | RANK METRIC SCORE | RUNNING ES | CORE ENRICHMENT || 1 | SMPDL3A | sphingomyelin phosphodiesterase acid like 3A [Source:HGNC Symbol;Acc:HGNC:17389] | 232 | 0.797 | 0.0664 | Yes |
| 2 | GALC | galactosylceramidase [Source:HGNC Symbol;Acc:HGNC:4115] | 478 | 0.669 | 0.1181 | Yes |
| 3 | SMPD2 | sphingomyelin phosphodiesterase 2 [Source:HGNC Symbol;Acc:HGNC:11121] | 821 | 0.564 | 0.1512 | Yes |
| 4 | PPT1 | palmitoyl-protein thioesterase 1 [Source:HGNC Symbol;Acc:HGNC:9325] | 1042 | 0.508 | 0.1880 | Yes |
| 5 | SMPD1 | sphingomyelin phosphodiesterase 1 [Source:HGNC Symbol;Acc:HGNC:11120] | 1230 | 0.471 | 0.2233 | Yes |
| 6 | HEXB | hexosaminidase subunit beta [Source:HGNC Symbol;Acc:HGNC:4879] | 1434 | 0.438 | 0.2539 | Yes |
| 7 | FUCA1 | alpha-L-fucosidase 1 [Source:HGNC Symbol;Acc:HGNC:4006] | 1506 | 0.428 | 0.2937 | Yes |
| 8 | NAGA | alpha-N-acetylgalactosaminidase [Source:HGNC Symbol;Acc:HGNC:7631] | 1674 | 0.402 | 0.3233 | Yes |
| 9 | GBA3 | glucosylceramidase beta 3 (gene/pseudogene) [Source:HGNC Symbol;Acc:HGNC:19069] | 1767 | 0.388 | 0.3573 | Yes |
| 10 | ASAH1 | N-acylsphingosine amidohydrolase 1 [Source:HGNC Symbol;Acc:HGNC:735] | 2201 | 0.338 | 0.3595 | Yes |
| 11 | PRKCD | protein kinase C delta [Source:HGNC Symbol;Acc:HGNC:9399] | 2237 | 0.335 | 0.3922 | Yes |
| 12 | GM2A | GM2 ganglioside activator [Source:HGNC Symbol;Acc:HGNC:4367] | 2299 | 0.327 | 0.4220 | Yes |
| 13 | MGST2 | microsomal glutathione S-transferase 2 [Source:HGNC Symbol;Acc:HGNC:7063] | 2583 | 0.292 | 0.4310 | Yes |
| 14 | HEXA | hexosaminidase subunit alpha [Source:HGNC Symbol;Acc:HGNC:4878] | 3025 | 0.247 | 0.4229 | Yes |
| 15 | PNLIPRP2 | pancreatic lipase related protein 2 (gene/pseudogene) [Source:HGNC Symbol;Acc:HGNC:9157] | 3268 | 0.224 | 0.4279 | Yes |
| 16 | GBA | glucosylceramidase beta [Source:HGNC Symbol;Acc:HGNC:4177] | 3327 | 0.219 | 0.4465 | Yes |
| 17 | SGPL1 | sphingosine-1-phosphate lyase 1 [Source:HGNC Symbol;Acc:HGNC:10817] | 3462 | 0.206 | 0.4579 | Yes |
| 18 | GLA | galactosidase alpha [Source:HGNC Symbol;Acc:HGNC:4296] | 4172 | 0.140 | 0.4177 | No |
| 19 | NEU1 | neuraminidase 1 [Source:HGNC Symbol;Acc:HGNC:7758] | 4486 | 0.114 | 0.4054 | No |
| 20 | NEU2 | neuraminidase 2 [Source:HGNC Symbol;Acc:HGNC:7759] | 5775 | 0.010 | 0.3064 | No |
| 21 | CYP1B1 | cytochrome P450 family 1 subfamily B member 1 [Source:HGNC Symbol;Acc:HGNC:2597] | 5925 | 0.000 | 0.2949 | No |
| 22 | SMPD3 | sphingomyelin phosphodiesterase 3 [Source:HGNC Symbol;Acc:HGNC:14240] | 6533 | -0.045 | 0.2525 | No |
| 23 | SMPDL3B | sphingomyelin phosphodiesterase acid like 3B [Source:HGNC Symbol;Acc:HGNC:21416] | 7293 | -0.102 | 0.2043 | No |
| 24 | CEL | carboxyl ester lipase [Source:HGNC Symbol;Acc:HGNC:1848] | 8375 | -0.184 | 0.1398 | No |
| 25 | ENPP2 | ectonucleotide pyrophosphatase/phosphodiesterase 2 [Source:HGNC Symbol;Acc:HGNC:3357] | 8442 | -0.189 | 0.1547 | No |
| 26 | NEU3 | neuraminidase 3 [Source:HGNC Symbol;Acc:HGNC:7760] | 9348 | -0.259 | 0.1119 | No |
| 27 | SPHK1 | sphingosine kinase 1 [Source:HGNC Symbol;Acc:HGNC:11240] | 12320 | -0.682 | -0.0467 | No |
| 28 | SMPD4 | sphingomyelin phosphodiesterase 4 [Source:HGNC Symbol;Acc:HGNC:32949] | 12702 | -0.866 | 0.0154 | No |
Table: GSEA details [plain text format]

  

Fig 2: GOBP\_MEMBRANE\_LIPID\_CATABOLIC\_PROCESS      
 Blue-Pink O' Gram in the Space of the Analyzed GeneSet

  

Fig 3: GOBP\_MEMBRANE\_LIPID\_CATABOLIC\_PROCESS: Random ES distribution      
 Gene set null distribution of ES for **GOBP\_MEMBRANE\_LIPID\_CATABOLIC\_PROCESS**
